# Community Dissemination of a Community-Engaged Research Project: Approaches and Lessons Learned

**DOI:** 10.1093/geroni/igaf122.1277

**Published:** 2025-12-31

**Authors:** Truphosa (Posa) Aswani, Wynfred Russell, Tetyana Shippee, Joseph Gaugler, Manka Nkimbeng

**Affiliations:** University of Washington, Seattle, Minnesota, United States; Strengthening African Resilience Towards Excellence, Brooklyn Park, Minnesota, United States; University of Minnesota, Minneapolis, Minnesota, United States; University of Minnesota, Minneapolis, Minnesota, United States; University of Minnesota School of Public Health, Minneapolis, Minnesota, United States

## Abstract

Beginning in September 2019, we conducted a dementia care needs and assets assessment with the African immigrant community in Minnesota. Project advisory board members informed the development of a community conversation guide. Findings from the community conversations were used to develop a survey that was subsequently implemented through various community partner organizations. As a community-based participatory research project, there was a project dissemination plan that included presentations to community and scientific audiences. Our presentation will describe a community-engaged approach to disseminating the findings of the needs and assets assessment with the African immigrant community. Community dissemination of the findings was done through diverse events, activities, and partnerships both in person and remotely. In-person presentation of the findings was done at partner churches, mosques, and community fairs, while remote presentations were done via social media and Zoom. Finally, we partnered with another project to disseminate key findings to the community in person and through booklets. Project findings have been disseminated to over 1000 members of the community. Project findings have also been disseminated to scientific audiences through three journal publications, with one more under review. Our approach to dissemination, while successful, has not been without challenges. As such, we will also present lessons learned in the process of disseminating project findings to the community.

